# SHR2285, the first selectively oral FXIa inhibitor in China: Safety, tolerability, pharmacokinetics and pharmacodynamics combined with aspirin, clopidogrel or ticagrelor

**DOI:** 10.3389/fphar.2022.1027627

**Published:** 2022-10-19

**Authors:** Tingting Ma, Yanli Dong, Lei Huang, Yuanxun Yang, Yan Geng, Fei Fei, Pinhao Xie, Yu Zhao, Hui Lin, Zeyu Yang, Yun Jin, Xitong Ju, Runbin Sun, Juan Li

**Affiliations:** ^1^ Phase I Clinical Trials Unit, Nanjing Drum Tower Hospital Clinical College of Jiangsu University, Nanjing, China; ^2^ Jiangsu Hengrui Pharmaceuticals Co.,Ltd., Lianyungang, China

**Keywords:** anticoagulants, coagulation factor inhibitor, dual antiplatelet therapy, pharmacodynamics, pharmacokinetics

## Abstract

**Purpose:** To evaluate the safety, tolerability, pharmacokinetics and pharmacodynamics of SHR2285, the first oral coagulation factor XIa (FXIa) inhibitor developed in China in combination with aspirin, clopidogrel or ticagrelor in healthy subjects.

**Methods:** This study was a single-center, randomized, double-blind, placebo-controlled (only SHR2285) design (NCT04945616). A total of 52 healthy subjects, 29 male and 23 female, were completed in this study. The subjects were divided into three groups: A, B and C, 16 subjects in group A [aspirin + clopidogrel + placebo or SHR2285 200 mg bid (1:3, 4 received placebo and 12 received SHR2285)] 16 subjects in group B [aspirin + clopidogrel + placebo or SHR2285 300 mg bid (1:3, 3 received placebo and 13 received SHR2285)] and 20 subjects in group C (aspirin + ticagrelor + placebo or SHR2285 300 mg bid (2:3, 8 received placebo and 12 received SHR2285)), respectively. All groups were administered orally for six consecutive days. Safety, tolerability, pharmacokinetics and pharmacodynamics parameters were assessed.

**Results:** 1) SHR2285 was well tolerated, and all adverse events were mild. There was no evidence of an increased risk of bleeding. 2) After 6 days of twice-daily administration, SHR2285 could reach a steady state. The mean half-life of SHR2285 in group A, group B and group C was 13.9 h, 14.5 h and 13.8 h, respectively. 3) SHR2285 markedly inhibited FXI activity and prolonged activated partial thromboplastin time (APTT). In group A, group B and group C, the mean maximum inhibition rate of FXI activity was 84.8%, 89.3% and 92.2% and the mean maximum prolongation of APTT was 2.08-fold, 2.36-fold and 2.26-fold, respectively.

**Conclusion:** These data suggest that SHR2285, a potential oral FXIa inhibitor, is expected to become a novel, safe and effective anticoagulant when combined with aspirin, clopidogrel or ticagrelor.

## Introduction

With the aging of the population and the change in people’s lifestyles and habits, thromboembolic diseases have increasingly become a major global health problem and the top-ranked cause of global population death (Abbafati et al., 2020). Thromboembolic conditions were estimated to account for one in 4 deaths worldwide in 2010 and are the leading cause of mortality. Ischemic heart disease, ischemic stroke and atrial fibrillation are all included in the global disease burden project (Wendelboe and Raskob, 2016).

The short-term use of dual antiplatelet therapy (DAPT) based on aspirin and platelet P2Y12 receptor inhibitors (commonly clopidogrel and ticagrelor used in China) is the cornerstone of the treatment of cardiac and systemic ischemic events in patients with coronary heart disease with an elevated risk of ischemia (Degrauwe et al., 2017; Wang et al., 2017). DAPT has been shown to reduce recurrent major ischemic events. However, there are still about 5.0%–8.7% of cardiovascular death or ischemic events and 8.3% stroke recurrence rate (Degrauwe et al., 2017; Pan et al., 2021), indicating the involvement of hypercoagulability. In addition, DAPT significantly increases the risk of severe bleeding.

In recent years, drugs targeting coagulation factors have become a new research hot spot for anticoagulant therapy. Non-vitamin K antagonist oral anticoagulants (NOACs) are also known as novel oral anticoagulants directly inhibiting Xa (such as rivaroxaban, apixaban and edoxaban) or factor IIa (such as dabigatran) are widely used in clinical (Levi, 2016). The most important complication of anticoagulant therapy is haemorrhage, which may be severe or even life-threatening, limiting its clinical application. Consequently, the research and development of new antithrombotic drugs are extremely important for the treatment of thrombotic diseases, and it is urgent to explore a safe and effective distinctive oral anticoagulant to meet the clinical demands.

Human FXI is a dimer composed of 607 amino acids (Emsley et al., 2010). It belongs to the trypsin-like serine protease factor and is necessary to maintain the endogenous pathway. FXI forms a complex with high molecular weight (HMW) kininogen and circulates in plasma. It is disconnected between Arg369 and Ile370 by FXIIa and thrombin, and finally transformed into the active form FXIa, which participates in the amplification effect of the coagulation cascade (Emsley et al., 2010). Genomic studies show that individuals with genetic defects in FXIa do not have spontaneous bleeding, and high levels of FXI are closely related to coronary artery diseases such as venous thrombosis and myocardial infarction; On the contrary, FXI deficiency does not have a great impact on physiological hemostasis (Key, 2014; Georgi et al., 2019; Wang et al., 2021). Severe FXI deficiency (10%–20% of the normal value) can protect against venous thrombosis and reduce the incidence of thrombosis (Salomon et al., 2008; Preis et al., 2017). Therefore, FXIa inhibitor is expected to become a new, safe and effective anticoagulant drug.

FXIa inhibitors currently under clinical development include monoclonal antibodies [Osocimab [BAY-1213790] (Weitz et al., 2020), Abelacimab [MAA868] (Koch et al., 2019)]antisense oligonucleotides [FXI-ASO (ISIS 416858)] (Büller et al., 2015) and small molecules [BMS-986177 (Gómez-Outes et al., 2017), BMS-962212 (Perera et al., 2018), ONO-7684 (Beale et al., 2021), EP-7041 (Pollack et al., 2020)]. This indicates that FXIa inhibitors have great potential for treating thrombotic diseases without a substantial risk of bleeding. However, these compounds are in phase I or phase II clinical trials.

SHR2285 is a small molecule compound that selectively inhibits human FXIa, independently developed by Jiangsu Hengrui Pharmaceutical Co., Ltd. It is the first oral inhibitor of coagulation factor XIa (FXIa) developed in China and a new potential antithrombotic drug. At present, systematic preclinical pharmacology, pharmacokinetics and toxicology studies have been completed, and animal tests have confirmed that it selectively inhibits human FXIa, reduces thrombosis weight, prolongates APTT and reduces bleeding risk. Up to now, SHR2285 has completed three clinical trials (including SHR2285-101, SHR2285-102 and SHR2285-104), all of which are phase I trials in healthy subjects (Perera et al., 2018; Chen et al., 2022).

Triple antithrombotic therapy with warfarin plus two antiplatelet agents is the standard of care after percutaneous coronary intervention (PCI) for patients with a trial fibrillation. Recently, most guidelines recommended both anticoagulation and dual antiplatelet therapy (triple therapy) (Cannon et al., 2018). However, these therapies are associated with a high risk of bleeding. In the first-in-human study of SHR2285 (Chen et al., 2022), no bleeding events of SHR2285 were reported, indicating that SHR2285 might be a potential choice of anticoagulants for patients with a trial fibrillation who underwent PCI and required a combination of dual antiplatelet therapy with the anticoagulant drug. Refer to most guidelines for recommendations (Degrauwe et al., 2017; Wang et al., 2017), SHR2285 may be combined with aspirin, clopidogrel, or ticagrelor in the phase II clinical study planned to be carried out, and the patients are given multiple times of medication for a long time. In SHR2285-104 study (data not disclosed), it was predicted that the effective dose of SHR2285 tablets was 200 mg bid and above. Therefore, the dose of SHR2285 in this study is 200 mg bid or 300 mg bid. Still, when combined with dual antiplatelet agents, subjects have increased safety risks, especially the possibility of increased risk of bleeding, as well as the potential of pharmacokinetic (PK) and pharmacokinetic (PD) interactions. Therefore, the objective of this study was to evaluate the safety, tolerability, PK and PD of SHR2285 tablets in combination with aspirin, clopidogrel or ticagrelor in healthy subjects.

## Methods

This study (NCT04945616) was consensus on ethical principles based on international ethical guidelines, including the declaration of Helsinki, the international ethical guidelines of the Council for International Organization of Medical Sciences (CIOMS), the International Council for Harmonisation of Technical Requirements for Pharmaceuticals for Human Use (ICH) guidelines, and other applicable laws and regulations. The study was reviewed and approved in writing by the ethics committee of Nanjing Drum Tower Hospital, the Affiliated Hospital of Nanjing University Medical School, and the research plan, plan amendment, informed consent and other relevant documents such as recruitment advertisement were provided to the ethics committee. This trial strictly followed the good clinical practice (GCP) issued by National Medical Products Administration (NMPA). All subjects provided written informed consent.

### Study design

The overall study was a single-center, randomized, double-blind, placebo (SHR2285 only) control design. A total of 52 healthy subjects were completed in this study, including 29 males and 23 females. The subjects were divided into groups A, B and C. 16 subjects were randomised according to a Randomization and trial supply management (RTSM) by a study doctor using a SAS software program in group A and group B (1:3, four received placebo and 12 received SHR2285), 20 subjects in group C (2:3, 8 received comfort 12 received SHR2285). Each subject can only participate in the trial of one dose group.

For group A, the dosing regimen was aspirin + clopidogrel + placebo or SHR2285 200 mg. Specifically, aspirin: 100 mg was taken orally every morning from day 1 to day 6; clopidogrel: 300 mg in the morning of the first day, 75 mg in the morning of the second day to the sixth day; placebo or SHR2285 (1:3): 200 mg orally every morning and evening until the morning of the sixth day.

For group B, the dosing regimen was aspirin + clopidogrel + placebo or SHR2285 300 mg. The use of aspirin and clopidogrel was the same as that of group A; Placebo or SHR2285: added the amount to 300 mg based on group A.

For group C, the administration regimen was aspirin + ticagrelor + placebo or SHR2285 300 mg. The use of aspirin, placebo or SHR2285 was the same as that in group B. Clopidogrel in group B was adjusted to ticagrelor: 180 mg orally in the morning of the first day, 90 mg in the morning and evening from the second day to the morning of the sixth day.

The subjects were administered continuously from day 1 to day 6 by oral administration with about 240 ml of normal temperature water. They were required to fast for at least 8 h before administration in the morning and take it 1 h (±10 min) before breakfast; the administration time in the evening is 12 h (+15 min) from the administration in the morning. The administration of group B and group C was initiated after the researchers and the sponsor evaluated the safety data of all subjects in group A at 48 h after the last dosing. During the whole trial period, it was forbidden to take tea, coffee and other beverages containing coffee and alcohol, eat grapefruit or grapefruit juice, and smoke. Required work and rest regularly and avoided strenuous exercise.

SHR2285 was identical to the placebo except for the drug number. The blind state must be maintained from the enrollment of subjects, the recording and evaluation of study results, the inspection of the study process to data management. All subjects, researchers, the sponsor’s investigator participating in the clinical evaluation, and the supervisors (CRA) participating in the study were not informed about which drugs the subjects received for the study. The blind remained unbroken.

PK blood collection points: the blood of each group was collected in the morning of day 1 before administration (within 1 h before administration), 4 h and 12 h after administration (before administration in the evening); in the morning of day 3, day 4, and day 5 before administration (within 1 h before administration), within 1 h before administration on the morning of the sixth day, and 0.5 h, 1 h, 1.5 h, 2 h, 2.5 h, 3 h, 4 h, 5 h, 6 h, 8 h, 10 h, 12 h, 15 h, 24 h, 36 h, and 48 h after administration. About 7 ml of venous blood was collected, and the plasma was collected for concentration determination of aspirin and its active metabolites (salicylic acid) and clopyridine (group A, group B), ticagrelor and its active metabolite AR-C124910XX (group C), SHR2285 and its active metabolite SHR164471.

PD blood collection points: the blood of each group was collected in the morning of the first day before administration (within 1 h before administration) and 4 h and 12 h after administration (before evening administration); the morning of the third, fourth, and fifth days before administration (within 1 h before administration), before administration on the morning of the sixth day (within 1 h before administration), and 1.5 h, 2 h, 2.5 h, 3 h, 4 h, 6 h, 8 h, 12 h, 24 h, 36 h, and 48 h after administration. About 6 ml of venous blood was collected for FXIa (about 3 ml) and coagulation function testing (about 3 ml).

### Subjects

Subjects were healthy volunteers aged 18–55 years, both male and female (No pregnant or breastfeeding; Contraception for at least 30 days before screening; The next menstrual period is at least five drug half-lives, about 5 days). The body mass index (BMI) of the subjects was 19–28 kg/m^2^, the male body weight was ≥50.0 Kg and <90.0 Kg and the female body weight was ≥45.0 Kg and <90.0 Kg; No smoker within 1 month before screening; The average daily alcohol intake in a week was less than 15 g. Strict contraception should be used during the trial and for 90 days after administration. The subjects could understand the research procedures and methods, voluntarily participate in the trial and sign written informed consent.

The subjects should not have any allergy to the suspected study drug or any related components, or have a history of abnormal coagulation function, high-risk bleeding risk and affecting drug absorption. Subjects were not allowed to take any prescription drugs, over-the-counter drugs and Chinese herbal medicine within 14 days before taking the study drug.

### Research objectives and indicators

The primary objective of this study was to evaluate the safety and tolerability of SHR2285 tablets combined with aspirin, clopidogrel or ticagrelor in healthy subjects, that is, the incidence and severity of adverse events (rated as mild, moderate and severe), including subjects’ main complaint, physical examination, vital signs, laboratory examination and ECG, *etc.*

The secondary objective was to evaluate 1) the pharmacokinetic (PK) of aspirin and its active metabolite salicylic acid, clopidogrel, SHR2285 and its active metabolite SHR164471 in group A and group B. In group C, the PK of aspirin and its active metabolite salicylic acid, ticagrelor and its active metabolite AR-C124910XX, SHR2285 and its active metabolite SHR164471 were measured. 2) The pharmacodynamics (PD) of aspirin plus clopidogrel or ticagrelor with or without SHR2285 tablets in healthy subjects. PD indexes included coagulation factor XIa (FXIa) activity, activated partial thromboplastin time (APTT), prothrombin time (PT), and international normalized ratio (INR). On the first day before administration, the measured values of coagulation FXI activity and coagulation function (APTT, PT, INR) were taken as the baseline.

### Data analysis and statistical methods

Full analysis set (FAS), safety analysis set: all subjects who have received the study drug at least once. PK concentration and parameter analysis set: all randomized subjects who have used the study drug and have at least one PK concentration or parameter data. PD analysis set: all randomized subjects who have used the test drug and have a pre-medication baseline and at least one post-medication PD evaluation data.

The classified data are summarized by frequency and percentage. Continuous data are summarized by subject number, mean, standard deviation, median, maximum and minimum. Blood drug concentration data and PK parameters were summarized by subject number, mean, standard deviation, median, maximum, minimum, coefficient of variation, geometric mean, geometric standard deviation and geometric coefficient of variation.

Based on the PK concentration analysis set, the blood drug concentration was listed and summarized by descriptive statistics according to the medication group at the planned time. The mean and median blood concentration-time curved and semi-logarithmic diagrams were drawn according to the medication. In addition, the actual blood sample collection time was used to draw the blood concentration-time curve and semi-logarithmic diagram of individual subjects.

Based on the actual sampling time, the PK parameters of each component were calculated as a non-compartmental model by WinNonlin (Certara United States Inc, version 8.1). The pharmacokinetic parameters after multiple administrations were investigated: C_max_, AUC_tau_, AUC_0-last_, AUC_0-inf_, T_max_, t_1/2_, CL_ss_/F, V_ss/_F, C_trough_ and C_average_. The main pharmacokinetic parameters such as AUC_0-last_, AUC_0-inf_ and C_max_ were tabulated and summarized by descriptive statistics.

After logarithmic conversion, the AUC_ss_ and C_max_ of each component in the SHR2285 group (aspirin + clopidogrel/ticagrelor + SHR2285) and placebo group (aspirin + clopidogrel/ticagrelor + placebo) were statistically analyzed by ANOVA model. The medication group was used as the independent variable. The geometric mean ratio of the corresponding PK parameter (SHR2285/placebo group) and the estimated value of its 90% confidence interval was obtained by taking the exponential conversion from the mean difference estimated by the model (SHR2285 group-placebo group) and its 90% confidence interval. SHR2285 and its metabolite SHR164471, aspirin/salicylic acid, clopidogrel, ticagrelor and its metabolite AR-C124910XX were determined by Frontage Laboratories Co., Ltd. (Shanghai, China) using high-performance liquid chromatography-tandem mass spectrometry (HPLC-MS, Sciex API 4000). The chromatographic column is WATERS CORTECS®T3 (50 mm × 4.6 mm, id 2.7 μm), column temperature is set at 40°C. Mobile phase A is ultrapure water (containing 0.1% formic acid), and mobile phase B is acetonitrile/water (90:10, containing 0.1% formic acid). The elution gradient is: 0–0.4 min, A-B (50:50), 0.4–0.8 min, A-B (5:95), 0.8–2 min, A-B (5:95), 2–2.4 (50:50), 2.4–3.5 min, A-B (50:50). The total time was 3.5 min and the flow rate was 1.0 ml/min. The ion source was ESI source at positive ion mode. The heating capillary temperature was 400°C; CAD was nine; curtain gas was 30; GS1 (N2) was 50; GS2 (N2) was 50; The method used was multiple reaction monitoring (MRM), and the ion reactions used for quantitative analysis were m/z 545.1→408.0 (SHR2285) and m/z 721.2→408.1 (SHR164471), respectively. The calibration ranges of SHR2285 and SHR164471 were 5 ng/ml (lower limit of quantification) to 5,000 ng/ml. The calibration model was linear regression, quality control samples (15–4,000 ng/ml) were determined with accuracies of 98.0%–104.5% and 100.9%–104.2%, precision of ≤6.2% and ≤6.6%, and recovery of 98.7%–103.6% and 97.3%–102.1%, respectively for both analytes.

The absolute value and relative baseline change percentage of coagulation factor XI (FXI) activity, APTT, PT, INR (placebo group, SHR2285 group) and planned sampling time points of healthy subjects after administration were summarized and plotted (if applicable). FXI activity was measured by the Institute of Blood Transfusion, Chinese Academy of Medical Sciences of China using coagulation method. APTT was measured by Guangzhou Kingmed Center For Clinical Laboratory Co., Ltd. (China) using the coagulation method. PT was counted on the coagulation analyzer, and INR was calculated through parameter calculation. PD parameters APTT, PT, INR and FXI activity were measured with validated assays at Guangzhou KingMed Diagnostics Group Co., Ltd. and the Institute of Blood Transfusion, Chinese Academy of Medical Sciences of China. APTT and PT was evaluated using Dade Actin FSL activated APTT reagent and Tromborel S reagent respectively, automated on the instrument Sysmex CS5100 with a within-batch and inter-batch precision of both not high than 5%, and INR was calculated based on PT test results. FXI activity was measured using a clotting method by Dade actin activated cephaloplastin reagent, automated on Sysmex CS 2000i Coagulation analyser. The result data are presented as a percentage of normal, and its calibration range was 6.25%–150.0%.

## Results

### Subject demographics

**FIGURE 1:**
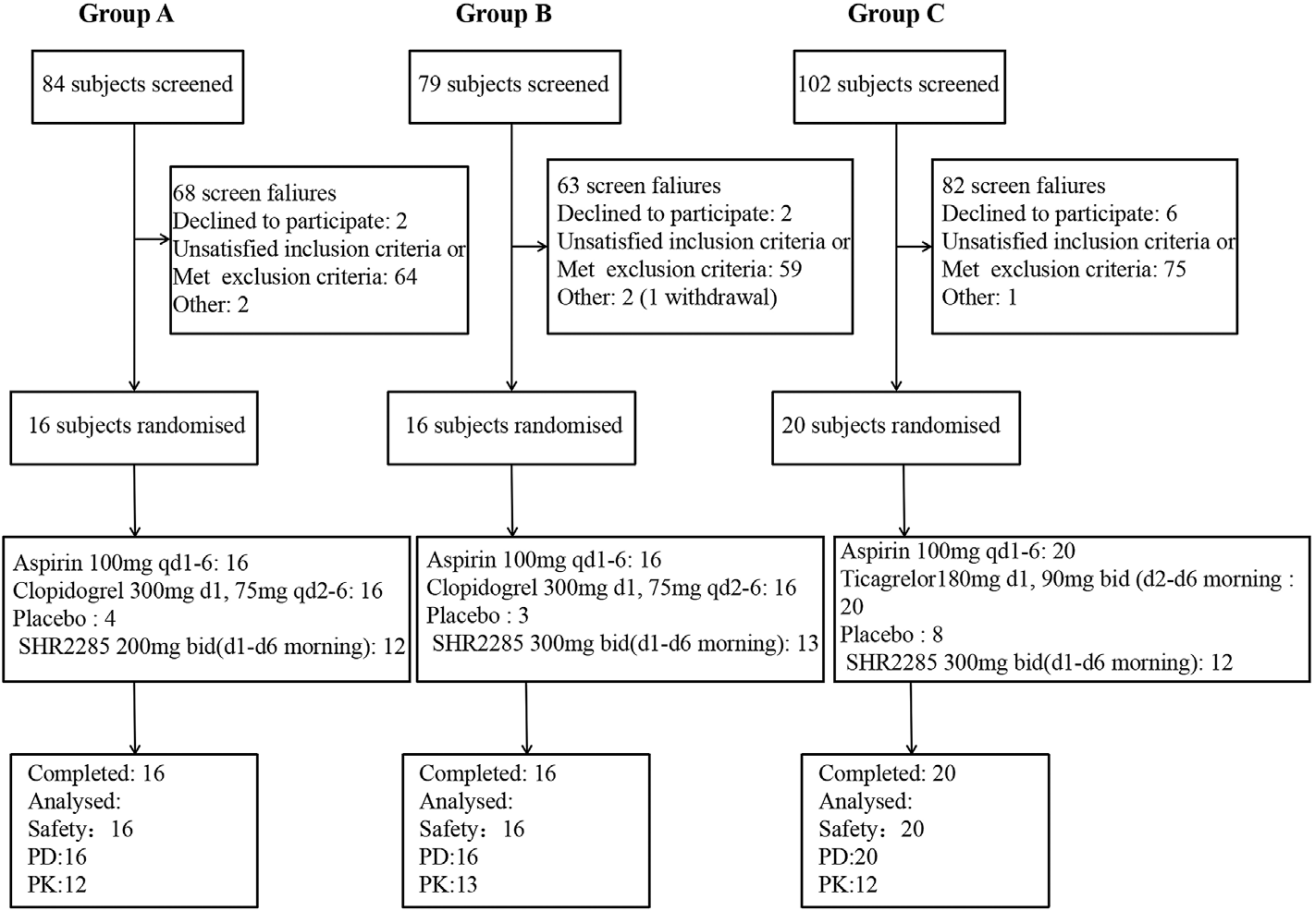
**(A)** Subject disposition of participants in group A of the study **(B)** Subject disposition of participants in group B of the study. **(C)** Subject disposition of participants in group C of the study. PD, pharmacodynamics; PK, pharmacokinetics; qd, once daily; bid, twice a day, once in the morning and once in the evening. There was unwilling to see an error in the randomization system, resulting in three placebos and 13 trial drugs in group **(B)** (One subject withdrew due to an adverse event, and the substitute subject accepted the drug randomization).

The study was conducted from 16 July 2021 to 16 November 2021. In group A and group B, all 32 subjects completed the study; in group C, all 20 subjects completed the study (Figure 1). All subjects were Chinese. The demographics of the subjects are shown in Table 1.

**TABLE 1:** Subject demographics.

	Treatment (placebo or SHR2285)				
	Placebo (A + B) n = 7	Placebo(C) n = 8	SHR2285 (A) n = 12	SHR2285(B) n = 13	SHR2285(C) n = 12
Age, years	26.4 (6.21)	27.3 (9.82)	36.0 (14.29)	33.1 (10.32)	29.3 (9.48)
Female, n (%)	3 (42.9)	4 (50.0)	7 (58.3)	4 (30.8)	5 (41.7)
Male, n (%)	4 (57.1)	4 (50.0)	5 (41.7)	9 (69.2)	7 (58.3)
Han nationality, n (%)	7 (100)	7 (87.5)	12 (100)	13 (100)	11 (91.7)
Height, cm	168.79 (5.322)	163.31 (5.548)	162.92 (6.704)	166.73 (11.088)	167.25 (10.947)
Weight, kg	67.13 (8.195)	62.01 (7.406)	62.66 (7.269)	63.22 (8.438)	67.36 (9.537)
BMI, kg/m^2^	23.5 (1.90)	23.2 (1.76)	23.6 (2.25)	22.7 (2.10)	24.0 (2.03)

Age, Height, Weight and BMI, are presented as mean (standard deviation). BMI, body massindex.

### Safety and tolerability

At least one treatment emergent adverse event (TEAE) occurred in 26 of 37 (70.3%) healthy subjects who received SHR2285 and 10 of 15 (66.7%) subjects who received placebo. The TEAEs of subjects are shown in Table 2.

**TABLE 2:** Summary of adverse events.

Term, n (%)	Placebo			SHR2285				Total n = 52
	A + B n = 7	C n = 8	Total n = 15	A n = 12	B n = 13	C n = 12	Total n = 37	
Epistaxis	0	0	0	2 (16.7)	0	0	2 (5.4)	2 (3.8)
Gingival bleeding	0	0	0	1 (8.3)	1 (7.7)	0	2 (5.4)	2 (3.8)
Oral Ulcer	1 (14.3)	0	1 (6.7)	1 (8.3)	2 (15.4)	0	3 (8.1)	4 (7.7)
Scratch	0	0	0	0	1 (7.7)	0	1 (2.7)	1 (1.9)
Subcutaneous hemorrhage	0	2 (25.0)	2 (13.3)	1 (8.3)	0	0	1 (2.7)	3 (5.8)
Diarrhea	0	0	0	0	2 (15.4)	0	2 (5.4)	2 (3.8)
Constipation	0	0	0	0	1 (7.7)	0	1 (2.7)	1 (1.9)
Ecchymosis	0	3 (37.5)	3 (20.0)	0	0	5 (41.7)	5 (13.5)	8 (15.4)
Rash	0	0	0	0	0	1 (8.3)	2 (2.7)	1 (1.9)
URTI	0	1 (12.5)	1 (6.7)	0	0	2 (16.7)	2 (5.4)	3 (5.8)
Abdominalburning sensation	0	0	0	0	0	1 (8.3)	1 (2.7)	1 (1.9)
Decreased Hb	0	1 (12.5)	1 (6.7)	2 (16.7)	1 (7.7)	0	3 (8.1)	4 (7.7)
Increased WBC	0	0	0	1 (8.3)	0	0	1 (2.7)	1 (1.9)
Increased N	0	0	0	1 (8.3)	0	0	1 (2.7)	1 (1.9)
Increased M	0	0	0	1 (8.3)	0	0	1 (2.7)	1 (1.9)
Fecal OB(+)	1 (14.3)	1 (12.5)	2 (13.3)	2 (16.7)	0	0	2 (5.4)	4 (7.7)
Urine OB(+)	0	1 (12.5)	1 (6.7)	0	0	0	0	1 (1.9)
Elevated ALT	0	0	0	0	1 (7.7)	0	1 (2.7)	1 (1.9)
Elevated TBil	0	1 (12.5)	1 (6.7)	0	1 (7.7)	0	1 (2.7)	2 (3.8)
Elevated Ca	0	0	0	0	1 (7.7)	0	1 (2.7)	1 (1.9)
Elevated TG	2 (28.6)	0	2 (13.3)	5 (41.7)	2 (15.4)	3 (25.0)	10 (27.0)	12 (23.1)
Hypercholesterolemia	0	0	0	0	1 (7.7)	0	1 (2.7)	1 (1.9)
Elevated UA	2 (28.6)	1 (12.5)	3 (20.0)	2 (16.7)	1 (7.7)	5 (41.7)	8 (21.6)	11 (21.2)
Elevated CK	0	1 (12.5)	1 (6.7)	0	0	0	0	1 (1.9)
ECG T wave changes	1 (14.3)	3 (37.5)	4 (26.7)	2 (16.7)	0	1 (8.3)	3 (8.1)	7 (13.5)
Prolonged QTc	0	0	0	0	0	1 (8.3)	1 (2.7)	1 (1.9)

URTI, upper respiratory tract infection; Hb, Hemoglobin; WBC, white blood cell count; N, neutrophil count; M, monocyte count; OB(+), occult blood positive; ALT, alanine aminotransferase; TBil, total bilirubin; Ca, blood calcium; TG, triglycerides; UA, serum uric acid; CK, creatine kinase; ECG, electrocardiogram; QTc, QTc interval of ECG. There were no serious adverse events during the entire study and no TEAEs, that led to treatment discontinuation or interruption.

Gingival bleeding and/or epistaxis occurred in three subjects in the SHR2285 dose group (A and B), but not in the placebo group. TEAE that occurred in or more of healthy subjects receiving SHR2285 compared with subjects treated with placebo included subcutaneous hemorrhage (2.7% vs. 13.3%), ecchymosis (13.5% vs. 20.0%), oral ulcer (8.1% vs. 6.7%), abdominal burning sensation (2.7% vs. 0), scratch (2.7% vs. 0), diarrhea (5.4% vs. 0), constipation (2.7% vs. 0), rash (2.7% vs. 0).

We also observed abnormalities in clinically relevant laboratory values in the SHR2285 dose group compared with the placebo group, including decreased hemoglobin (8.1% vs. 6.7%), fecal occult blood positive (5.4% vs. 13.3%), urine occult blood positive (0 vs. 6.7%), elevated alanine aminotransferase (2.7% vs. 0), elevated total bilirubin (2.7% vs. 6.7%), elevated triglycerides (27% vs. 13.3%), elevated serum uric acid (21.6% vs. 20.0%), elevated blood calcium (2.7% vs. 0), hypercholesterolemia (2.7% vs. 0), elevated creatine kinase (0 vs. 6.7%), electrocardiogram T wave abnormal (8.1% vs. 26.7%), prolonged QTc interval of electrocardiogram (2.7% vs. 0).

All TEAE in healthy subjects were mild. AEs in the SHR2285 group did not increase apparently compared with the placebo group, suggesting that the AEs may be caused by the combination of aspirin, clopidogrel or ticagrelor, indicating that the combination of SHR2285 with dual antiplatelet treatment did not increase the TEAE. The challenge of FXIa inhibitor combined with dual antiplatelet therapy is the risk of bleeding. In this trial, three subjects with transient mild gingival bleeding and epistaxis in SHR2285 dose group A and B compared with the placebo group. The AE of bleeding mentioned in SHR2285 300 mg dose group C was only ecchymosis, which occurred in five of 12 subjects, compared with three of eight subjects with placebo. No higher risk of bleeding was observed in group C than in the placebo group. These data suggest that it may be an adverse reaction of aspirin combined with clopidogrel or ticagrelor, and less likely to be related to SHR2285. Importantly, there was no evidence of a severe hemorrhage risk or serious adverse event (SAE). All events were resolved without treatment. None of the healthy subjects died or stopped taking TEAE.

### Pharmacokinetics

**FIGURE 2:**
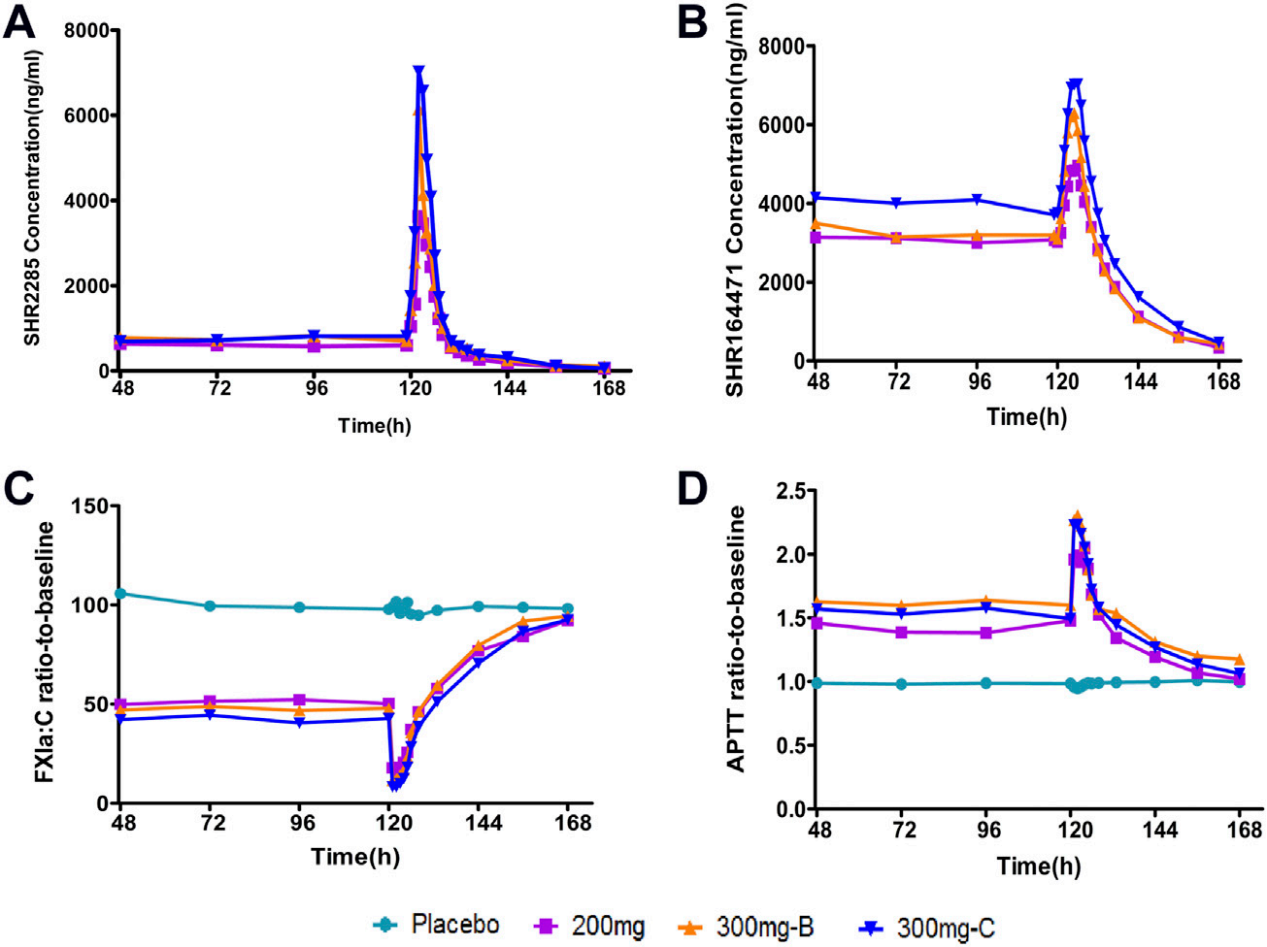
Mean PK and PD data following administration of multiple doses of SHR2285 for 6 days **(A)** Time course of mean SHR2285 concentrations; **(B)** Time course of mean SHR164471 (metabolite of SHR2285) concentrations **(C)** Time course of mean ratio-to-baseline values for FXIa:C; **(D)** Time course of mean ratio-to-baseline values for APTT. APTT, activated partial thromboplastin time; FXIa:C, factor XIa coagulation activity; PD, pharmacodynamics; PK, pharmacokinetics.

Combined with aspirin, clopidogrel or ticagrelor, three SHR2285 dose groups were administered 200 mg or 300 mg SHR2285 bid for six consecutive days, SHR2285 and its active metabolite SHR164471 in plasma reached a steady-state (Figures 2A,B). The mean plasma concentration of SHR2285 in each group peaked at approximately 1.5 h after the last administration on day 6 and then gradually decreased to baseline levels by 48 h (Figure 2A).

In the three groups, the peak of SHR2285 in plasma was faster, T_max_ was about 1.5 h. The mean t_1/2_ for SHR2285 after Day 6 was roughly the same (13.8–14.5 h). The peak of SHR164471 in plasma was later than the prototype, T_max_ was 2.5–4 h, and the t_1/2_ was 12.3–14.3 h (Tables 3, 4).

**TABLE 3:** SHR2285 Pharmacokinetic parameters.

	Treatment (SHR2285)		
	Group A n = 12	Group B n = 13	Group C n = 12
C_max_, ng/mL	3,780 (28.8)	6,020 (36.7)	7,290 (16.1)
T_max_, h	1.5 (1.5–2.5)	1.5 (1.5–2.0)	1.5 (1.5–2.0)
AUC_tau_, h*ng/mL	14,300 (26.4)	19,700 (33.7)	25,200 (22.9)
AUC_0-last_, h*ng/mL	19,400 (32.5)	28,100 (31.0)	33,800 (21.5)
AUC_0-inf_, h*ng/mL	19,600 (33.0)	29,500 (27.5)	34,900 (24.9)
t_1/2_, h	13.9 (5.56)	14.5 (5.43)	13.8 (7.02)
CL_ss_/F, L/h	14.8 (3.68)	15.7 (4.15)	12.2 (2.75)
V_ss_/F, L	283 (87.8)	343 (213)	227 (76.4)
C_trough_, ng/mL	337 (43.9)	501 (40.1)	505 (29.1)
C_average_, ng/mL	1,190 (26.4)	1,650 (33.7)	2,100 (22.9)

C_max_, AUC_tau_, AUC_0-last_ and AUC_0-inf_ are presented as geometric mean (GCV), T_max_ as median (range), t_1/2,_ CL_ss_/F and V_ss_/F as mean (standard deviation).

GCV, geometric coefficient of variation; C_max_, maximum concentration; T_max_, time to reach maximum concentration; AUC_tau_, area under the plasma concentration-time curveat steady-state (one dose interval); AUC_0-last_, area under the plasma concentration-time curve from time 0 to last time of quantifiable concentration; AUC_0-inf_, area under the plasma concentration-time curve from time 0 extrapolated to infinite time; t_1/2_, elimination half-life; CL_ss_/F, steady state clearance rate of oral administration; V_ss_/F, apparent volume of distribution; C_trough_, valley concentration; C_average_, steady-state average concentration.

**TABLE 4:** SHR164471 Pharmacokinetic parameters.

	Treatment (SHR2285)		
	Group A n = 12	Group B n = 13	Group C n = 12
C_max_, ng/mL	5,030 (18.7)	6,330 (25.1)	7,050 (34.3)
T_max_, h	4.0 (1.5–4.0)	2.5 (2.0–4.0)	3.0 (2.5–4.0)
AUC_tau_, h*ng/mL	43,200 (23.3)	48,000 (24.6)	57,600 (37.2)
AUC_0-last_, h*ng/mL	76,000 (30.6)	80,800 (30.5)	102,000 (42.8)
AUC_0-inf_, h*ng/mL	82,100 (33.6)	83,800 (31.0)	111,000 (40.8)
t_1/2_, h	13.1 (2.52)	12.3 (2.52)	14.3 (3.69)
CL_ss_/F, L/h	6.29 (1.53)	8.69 (2.08)	7.36 (3.12)
V_ss_/F, L	116 (20.7)	151 (29.5)	165 (119)
C_trough_, ng/mL	2,250 (32.0)	2,220 (26.2)	2,830 (45.7)
C_average_, ng/mL	3,600 (23.3)	4,000 (24.6)	4,800 (37.2)

C_max_, AUC_tau_, AUC_0-last_ and AUC_0-inf_ are presented as geometric mean (GCV), T_max_ as median (range), t_1/2,_ CL_ss_/F and V_ss_/F as mean (standard deviation).

GCV, geometric coefficient of variation; C_max_, maximum concentration;T_max_, time to reach maximum concentration; AUC_tau_, area under the plasma concentration-time curveat steady-state (one dose interval); AUC_0-last_, area under the plasma concentration-time curve from time 0 to last time of quantifiable concentration; AUC_0-inf_, area under the plasma concentration-time curve from time 0 extrapolated to infinite time; t_1/2_, elimination half-life; CL_ss_/F, steady state clearance rate of oral administration; V_ss_/F, apparent volume of distribution; C_trough_, valley concentration; C_average_, steady-state average concentration.

Dose proportionality in SHR2285 exposure in group A and group B was observed as follows: C_max_ and AUC_tau_ values increased about 1.6- and 1.38-fold with a 1.5-fold increase in dose, respectively (geometric means of 3,780–6,020 ng/ml for C_max_ and 14,300–19700 h*ng/mL for AUC_tau_; Table 3). Correspondingly, SHR164471 exposure (C_max_ and AUC_tau_) increased by 1.3- and 1.1- fold, respectively (Table 4).

**FIGURE 3:**
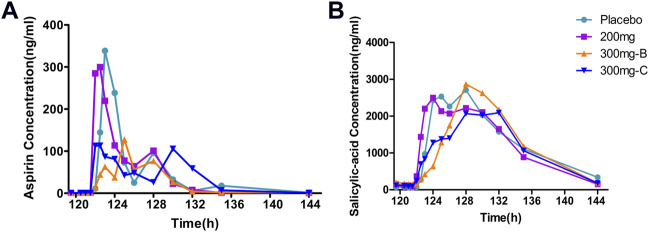
Mean PK data following administration of multiple doses of aspirin for 6 days **(A)** Time course of mean aspirin concentrations; **(B)** Time course of mean salicylic acid (metabolite of aspirin) concentrations. PK, pharmacokinetics.

Aspirin was administered once a day for six consecutive days. The median T_max_ of aspirin in groups A, B, C and placebo (A + B, C) were similar, which were 4.5 h, 6.0 h, 7.0 h, 8.0 h and 4.0 h, respectively (Supplementary Table S1), which was in line with the PK characteristics of delayed absorption of enteric-coated tablets in the drug instructions (Figure 3). The T_max_ of its active metabolite salicylic acid was slightly later than that of the prototype, with a median of 8.0 h, 8.0 h, 9.0 h, 8.0 h and 5.0 h, respectively (Supplementary Table S2). It was similar to the prototype, and T_max_ had a certain degree of inter-individual variation (Figure 3). The PK exposure (AUC_tau_, C_max_) of salicylic acid in the SHR2285 group and placebo group were similar, and no apparent difference was found between the two groups (Supplementary Tables S1, S2, S6).

**FIGURE 4:**
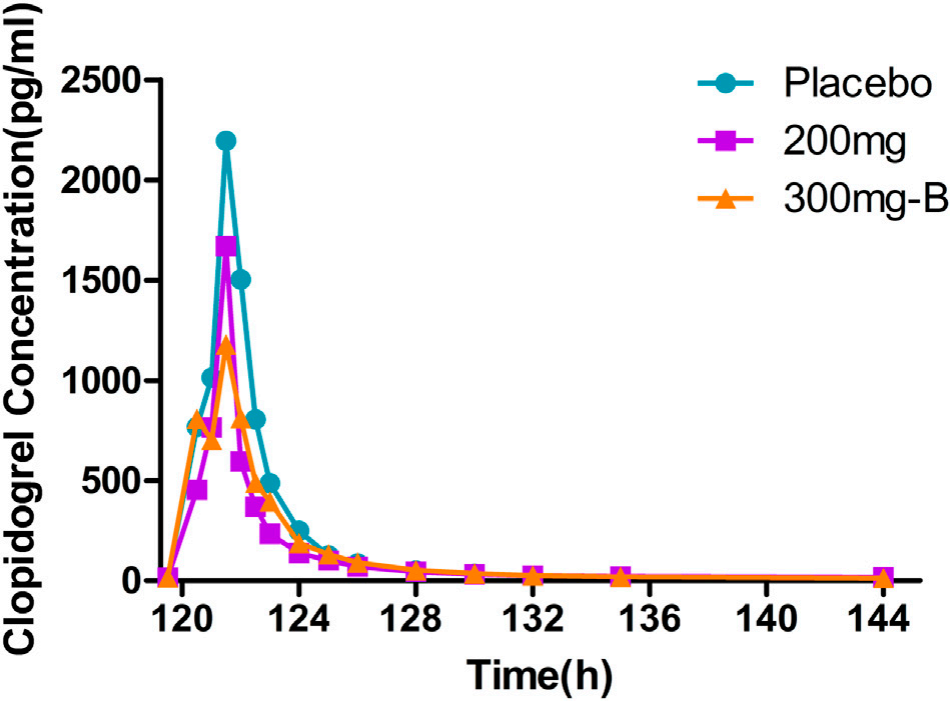
Mean PK data following administration of multiple doses of clopidogrel for 6 days. Time course of mean clopidogrel concentrations. PK, pharmacokinetics.

The peak of clopidogrel T_max_ in plasma was fast, ranging from one to 1.5 h. The clopidogrel exposure (C_max_, AUC) of group A and placebo were similar and slightly lower than that of group B with extremely high exposure in some individuals (Supplementary Tables S3, S6, Figure 4). CYP2C19 was involved in forming the active metabolite and intermediate metabolite 2-oxygen-clopidogrel, which is suspected to be related to gene polymorphism.

**FIGURE 5:**
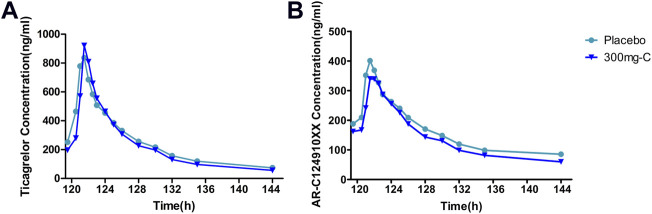
Mean PK data following administration of multiple doses of ticagrelor for 6 days **(A)** Time course of mean ticagrelor concentrations; **(B)** Time course of mean AR-C124910XX (metabolite of ticagrelor) concentrations. PK, pharmacokinetics.

Six days after administration, ticagrelor and its metabolite AR-C124910XX in plasma reached a steady state, and T_max_ peaked rapidly, ranging from 1.5 to 1.75 h. PK exposure (AUC_tau_, C_max_) of the SHR2285 dose group and placebo group were similar, and no noticeable difference was found between the groups (Supplementary Tables S4–S6, Figure 5).

### Pharmacodynamics

Following repeated administration of SHR2285 200 mg or 300 mg, the inhibition of FXI activity in the SHR2285 group was apparently higher than that in the placebo group (Table5), and there was no evident change in the inhibition level of FXI activity in the placebo group (Figure 2C).

**TABLE 5:** Pharmacodynamics indexes.

	Treatment (placebo or SHR2285)				
	Placebo (A + B) n = 7	Placebo(C) n = 8	SHR2285 (A) n = 12	SHR2285 (B) n = 13	SHR2285 (C) n = 12
FXIa:C					
Inhibition maximum	8.07 (3.37)	14.6 (6.88)	84.8 (4.66)	89.3 (3.85)	92.2 (1.66)
Inhibition average	-0.33 (4.88)	5.6 (7.06)	62.2 (6.53)	63.8 (5.50)	70.0 (5.33)
APTT					
RTB maximum	1.08 (0.0877)	1.04 (0.0372)	2.08 (0.199)	2.36 (0.224)	2.26 (0.125)
RTB average	0.988 (0.0619)	0.976 (0.0599)	1.67 (0.132)	1.78 (0.115)	1.76 (0.0866)

FXIa:C and APTT, values are presented as mean (standard deviation).

Percentage inhibition values are calculated using the percentage from baseline values (100% - FXI, activity%/baseline FXI, activity). FXIa:C, factor XIa, coagulation activity; APTT, activated partial thromboplastin time; RTB, ratio-to-baseline.

In the 200 mg SHR2285 group, there was a mean minimum on treatment ratio-to-baseline of 15.2%, corresponding to a mean maximum on treatment percentage inhibition vs baseline of 84.8%. The mean of the maximum inhibition rate of FXI activity in groups B and C were 89.3% and 92.2%, respectively (Table 5). The maximum inhibition of FXI activity occurred at 2 h, 1.5 h and 1.5 h (median) after administration; The FXI activity of the SHR2285 group gradually returned to the baseline level 48 h after administration (Figure 2C).

**FIGURE 6:**
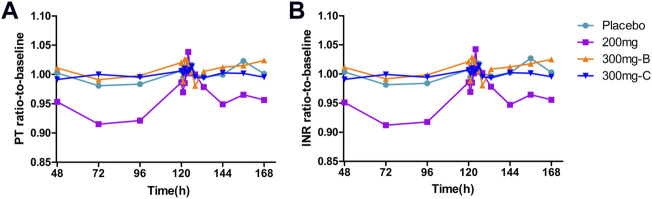
Mean PD data following administration of multiple doses of SHR2285 for 6 days **(A)** Time course of mean ratio-to-baseline values for PT; **(B)** Time course of mean ratio-to-baseline values for INR. PT, prothrombin time; INR, international normalized ratio; PD, pharmacodynamics.

Similar to the observed inhibitory effect of FXI activity, the APTT in the SHR2285 group was apparently more prolonged than that in the placebo group after repeated administration of 200 mg SHR2285 tablets in healthy subjects. There was almost no APTT prolongation in the placebo group (Figure 2D). The mean of the maximum APTT prolongation in the three SHR2285 dose groups were 2.08-, 2.36- and 2.26-fold, which appeared at 2 h, 1.5 h and 1.5 h (median), respectively (Table 5), and gradually returned to the baseline level at 48 h after administration. SHR2285 did not affect PT or INR (Figure 6).

The above data showed that the inhibition level of FXI activity was consistent with the increasing trend of plasma exposure of SHR2285 and its metabolite SHR164471, and they were positively correlated. In contrast, the correlation between APTT prolonged and plasma exposure of SHR2285 and SHR164471 was slightly weak.

### Pharmacokinetics and pharmacodynamic relationship

**FIGURE 7:**
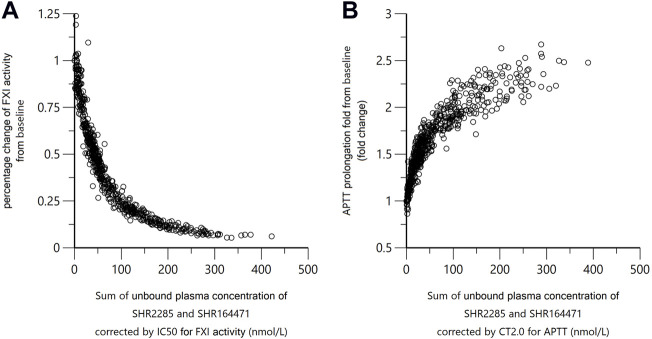
Correlation of SHR2285/SHR164471 unbound plasma concentrations with inhibition of FXI activity and APTT prolongation. In panel **(A)**, the *x*-axis indicates sum of unbounded plasma concentration of SHR2285 and SHR164471*IC50 of SHR2285 on FXI activity/IC50 of SHR164471 on FXI activity. In panel **(B)**, the *x*-axis represents the sum of unbounded plasma concentration of SHR2285 and SHR164471*CT2.0 of SHR2285 on APTT/CT2.0 of SHR164471 on APTT. The plasma-protein binding rate was measured using rapid equilibrium dialysis. Unbound plasma concentration = total plasma concentration*(1–protein binding%).

As the *in vitro* PD study showed that both SHR2285 and its main metabolite SHR164471 inhibited human FXI activity and prolonged the APTT, FXI activity and APTT in the study were plotted against the sum of SHR2285/SHR164471 unbound plasma concentration (Figure 7). There is an obvious positive correlation between pharmacokinetics and inhibition of FXI activity and APTT prolongation.

## Discussion

The emergence of antithrombotic drugs with different action mechanisms provides more choices for preventing and treating thrombotic diseases. Although the commonly used antithrombotic drugs have significant effects, some deficiencies still exist, such as adverse reactions, including bleeding, osteoporosis and thrombocytopenia, and undesired drug interactions. Therefore, the research and development of new antithrombotic drugs are crucial for treating thrombotic diseases. Severe FXI deficiency reduces the incidence of thrombosis without the risk of major bleeding, which provides a new strategy for FXIa inhibition as an anticoagulant therapy.

The PD parameters of SHR2285 in this clinical trial were similar to several developed FXI inhibitors. The phase II clinical trial data of antisense oligonucleotide FXI-ASO showed that subcutaneous injection of 300 mg FXI-ASO reduced FXI activity in patients undergoing total knee arthroplasty by 80%, APTT prolonged by 1.4 imes, and venous thromboembolism incidence rate was 4%, significantly lower than that in the enoxaparin group. However, it works slowly and may cause thrombocytopenia and an increased risk of postoperative bleeding (Büller et al., 2015). Some FXI inhibitors are administered intravenously; this invasive way of administration increases the adverse events of the infusion reaction and also limits the place of use, preferably in the hospital (Perera et al., 2018; Pollack et al., 2020; Weitz et al., 2020). Moreover, the risk of bleeding and the prevention of venous thromboembolism is not more effective than existing anticoagulants (Weitz et al., 2020). Highly selective oral FXIa inhibitors showed rapid onset and offset, the maximum inhibition rate of FXI activity is more than 90%, and the extension multiple of APTT is as high as 2.78 times (Gómez-Outes et al., 2017; Beale et al., 2021). In the SHR2285-101 study, the maximum FXI inhibition was 60.92%, and the APTT pro longed 1.52 times, slightly inferior to the PD of SHR2585 in this study (different dosage forms). In the first-in-human study of SHR2285, no bleeding events were reported, suggesting that SHR2285 may be a potential anticoagulant option for patients who underwent PCI and required DAPT in combination with anticoagulants (Chen et al., 2022). However, the antithrombotic effect and bleeding risk after FXIa inhibitor combined with the existing standard dual antiplatelet therapy, the standard treatment of myocardial infarction and stroke recommended by the international and China, has not been studied.

In this study, SHR2285 was well tolerated in healthy subjects. All randomized subjects completed the study as planned without any significant tolerance problems. All adverse events observed were mild and resolved without intervention. The incidence of AEs was similar in the placebo and SHR2285 dose groups. Importantly, there is no evidence of a risk of massive bleeding after combined administration, which remains a common problem of existing anticoagulant therapy. The mild bleeding events observed in the study may be the drug reaction of dual antiplatelet. The PK parameters of SHR2285 were comparable with other small molecule FXIa inhibitors such as BMS-986177, BMS-962212, ONO-7684 and EP-7041 (Gómez-Outes et al., 2017; Perera et al., 2018; Pollack et al., 2020; Beale et al., 2021). The pharmacokinetics of SHR2285 and its main active metabolite were well characterized. The t_1/2_ of SHR2285 and its active metabolite SHR164471 is 12.3–14.5 h, similar to the SHR2285-101 study (Chen et al., 2022), which supports oral administration twice a day, both inside and outside the hospital. In this study, C_max_ and AUC of SHR2285 were apparent higher than 101 study, and T_max_ was significantly apparent. In this study, SHR2285 was a solid dispersible tablet, and SHR2285-101 was a nanocrystalline preparation. It may be that different dosage forms lead to different absorption and bioavailability. PD studied in this trial included APTT, FXI activity and PT/INR. The increasing exposure could result in the prolongation of APTT and the decrease of FXI activity but had no effect on PT/INR, which may explain why SHR2285 does not increase the risk of bleeding.

Previous studies have suggested that anticoagulant drugs (including vitamin K antagonists, factor Xa inhibitors, *etc.*) combined with dual antiplatelet drugs showed a trend of reducing thrombotic events (not statistically significant), but it was also accompanied by an increased risk of bleeding, which was limited in clinical application (Lou et al., 2018). Therefore, to reduce the occurrence of thromboembolism without increasing the risk of bleeding, we can choose the treatment scheme of DAPT combined with FXIa (a new anticoagulant) to meet the clinical needs better. The relationship between SHR2285 and the prevention of thromboembolic events needs to be determined in subsequent phase II and III clinical trials.

In conclusion, SHR2285 significantly inhibited the activity of FXI in a dose-dependent manner. When combined with aspirin, clopidogrel or ticagrelor, it was well tolerated in healthy subjects without apparent safety or tolerance issues. There was no significant effect on PT or INR, and there was no evidence of an increased risk of bleeding. These results suggest that the FXIa inhibitor represented by SHR2285 is expected to be an effective and safe oral anticoagulant for combined dual antiplatelet therapy.

## Data Availability

The raw data supporting the conclusions of this article will be made available by the authors, without undue reservation.
