# Supplementary material for: SHR2285, the first selectively oral FXIa inhibitor in China: Safety, tolerability, pharmacokinetics and pharmacodynamics combined with aspirin, clopidogrel or ticagrelor
Source: Front Pharmacol. 2022 Oct 19;13:1027627. doi: 10.3389/fphar.2022.1027627 (PMC9626527; doi:10.3389/fphar.2022.1027627)
Supplement: Supplementary file 1 [file DataSheet1.docx]

**TABLE S1 Aspirin Pharmacokinetic parameters**

|  | **Treatment (placebo or SHR2285)** | | | | |
| --- | --- | --- | --- | --- | --- |
|  | **Placebo(A+B)**  **n=7** | **Placebo(C)**  **n=8** | **SHR2285 (A)**  **n=12** | **SHR2285(B) n=13** | **SHR2285(C) n=12** |
| **C_max_, ng/ml** | 211(441.1) | 565(93.8) | 416(128.1) | 189(101.5) | 241(129.3) |
| **T_max_, h** | 8.0(3.0-23.9) | 4.0(2.5-15.0) | 4.5(2.0-8.0) | 6.0(2.5-10.0) | 7.0(2.0-12.0) |
| **AUC_tau_, h*ng/mL** | -- | 1130(12.0) | 916(53.2) | -- | -- |

**--,** The number of effective cases is less than 50%, which is not included in the statistical analysis.

**TABLE S2 Salicylic acid Pharmacokinetic parameters**

|  | **Treatment (placebo or SHR2285)** | | | | |
| --- | --- | --- | --- | --- | --- |
|  | **Placebo(A+B)**  **n=7** | **Placebo(C)**  **n=8** | **SHR2285 (A)**  **n=12** | **SHR2285(B) n=13** | **SHR2285(C) n=12** |
| **C_max_, ng/ml** | 4260(35.5) | 5060(21.3) | 4350(44.3) | 2910(93.3) | 3940(54.8) |
| **T_max_, h** | 8.0(4.0-23.9) | 5.0(4.0-15.0) | 8.0(2.5-12.0) | 8.0(5.0-23.8) | 9.0(3.0-24.0) |
| **AUC_tau_, h*ng/mL** | 25300(32.6) | 31100(22.7) | 27100(31.7) | 25500(62.6) | 23600(25.9) |

**TABLE S3 Clopidogrel Pharmacokinetic parameters**

|  | **Treatment (placebo or SHR2285)** | | |
| --- | --- | --- | --- |
|  | **Placebo (A+B) n=7** | **SHR2285(A) n=12** | **SHR2285(B) n=13** |
| C_max_, pg/ml | 1000(272.0) | 1160(128.5) | 1260(90.6) |
| T_max_, h | 1.0(0.5-1.5) | 1.5(0.5-1.5) | 1.5(0.5-3.0) |
| AUC_tau_, h*pg/mL | 1970(210.8) | 1900(109.3) | 2490(77.1) |

**TABLE S4 Ticagrelor Pharmacokinetic parameters**

|  | **Treatment (placebo or SHR2285)** | |
| --- | --- | --- |
|  | **Placebo (C) n=8** | **SHR2285(C) n=12** |
| C_max_, ng/ml | 885(16.8) | 938(28.4) |
| T_max_, h | 1.5(1.0-1.5) | 1.75(1.5-2.0) |
| AUC_tau_, h*ng/mL | 4430(21.9) | 4120(33.7) |

**TABLE S5 AR-C124910XX Pharmacokinetic parameters**

|  | **Treatment (placebo or SHR2285)** | |
| --- | --- | --- |
|  | **Placebo (C) n=8** | **SHR2285(C) n=12** |
| C_max_, ng/ml | 389(35.4) | 367(28.1) |
| T_max_, h | 1.5(1.0-2.5) | 1.75(1.5-2.5) |
| AUC_tau_, h*ng/mL | 2490(38.3) | 2280(26.4) |

**TABLE S6 Comparison of PK parameters (PKPs) of combination (aspirin, salicylic acid, clopidogrel, ticagrelor and AR-C124910XX) in SHR2285 group and placebo group**

| **Analyte** | **Parameter** | **Group** | **n(SHR2285/ placebo)** | **GLSMR** | **90% CI** |
| --- | --- | --- | --- | --- | --- |
| Aspirin | C_max_, ng/ml | A/ placebo | 12/7 | 1.97 | (0.788,4.93) |
|  | AUCtau, h*ng/mL | A/ placebo | 12/7 | - | - |
|  | Cmax, ng/ml | B/ placebo | 13/7 | 0.897 | (0.363,2.21) |
|  | AUCtau, h*ng/mL | B/ placebo | 13/7 | - | - |
|  | Cmax, ng/ml | C/ placebo | 12/8 | 0.425 | (0.205,0.881) |
|  | AUCtau, h*ng/mL | C/ placebo | 12/8 | - | - |
| Salicylic acid | C_max_, ng/ml | A/ placebo | 12/7 | 1.02 | (0.634,1.65) |
|  | AUCtau, h*ng/mL | A/ placebo | 12/7 | 1.07 | (0.750,1.53) |
|  | Cmax, ng/ml | B/ placebo | 13/7 | 0.683 | (0.426,1.10) |
|  | AUCtau, h*ng/mL | B/ placebo | 13/7 | 1.01 | (0.710,1.43) |
|  | Cmax, ng/ml | C/ placebo | 12/8 | 0.779 | (0.558,1.09) |
|  | AUCtau, h*ng/mL | C/ placebo | 12/8 | 0.759 | (0.626,0.920) |
| Clopidogrel | C_max_, ng/ml | A/ placebo | 12/7 | 1.16 | (0.506,2.67) |
|  | AUCtau, h*ng/mL | A/ placebo | 12/7 | 0.962 | (0.458,2.02) |
|  | Cmax, ng/ml | B/ placebo | 13/7 | 1.26 | (0.557,2.87) |
|  | AUCtau, h*ng/mL | B/ placebo | 13/7 | 1.26 | (0.607,2.62) |
| Ticagrelor | Cmax, ng/ml | C/ placebo | 12/8 | 1.06 | (0.876,1.28) |
|  | AUCtau, h*ng/mL | C/ placebo | 12/8 | 0.929 | (0.739,1.17) |
| AR-C124910XX | Cmax, ng/ml | C/ placebo | 12/8 | 0.943 | (0.742,1.20) |
|  | AUCtau, h*ng/mL | C/ placebo | 12/8 | 0.916 | (0.718,1.17) |

GLSM, Geometric least squares mean ratios, CI confidence interval.

**-,** The number of effective cases is less than 50%, which is not included in the statistical analysis.
